# Toward Personalized Cell Therapies by Using Stem Cells 2013

**DOI:** 10.1155/2013/534047

**Published:** 2013-12-29

**Authors:** Ken-ichi Isobe, Herman S. Cheung, Ji Wu

**Affiliations:** ^1^Department of Immunology, Nagoya University Graduate School of Medicine, 65 Turumai-cho, Showa-ku, Nagoya, Aichi 466-8550, Japan; ^2^Biomedical Engineering Department, College of Engineering, University of Miami, Coral Gables, FL 33146, USA; ^3^Key Laboratory for the Genetics of Developmental and Neuropsychiatric Disorders, Ministry of Education, Bio-X Institutes, Shanghai Jiao Tong University, Shanghai 200240, China; ^4^School of Life Sciences and Biotechnology, Shanghai Jiao Tong University, Shanghai, China

The generation of induced pluripotent stem cells (iPSCs) from somatic cells by retrovirus transfection encoding Oct4, Sox2, cMyc, and Klf4 established a major landmark in the field of stem cell biology as it allows the establishment of patient-specific pluripotent cells. However, tumor formation and immunogenicity have been suggested if patients own iPSCs or differentiated cells are transplanted back to the patient. T. d. S. Fernandez et al. discussed why hiPSCs have the potential to induce tumors in host. Then they review the potential use of hiPSCs in clinical applications for cancer. In order to avoid oncogenic transformation, R. O'Doherty et al. reviewed the nonviral methods to induce pluripotency. S. Thanasegaran et al. clearly showed that iPSCs and differentiated tissue cells from iPSCs, which were established from retroviruses, had no immunogenicity by transplantation into syngeneic mice. Thus iPSCs have great possibility to treat many diseases such as neurological disorders summarized by N. Jongkamonwiwat and P. Noisa.

MSCs from bone marrow, skin, and adipose tissue have been used for the model experiments of stem cell therapy for many diseases. H. Kim et al., review the stem cell therapy in bladder dysfunction especially bladder outlet obstruction. Y. Gao et al. first isolated MSC from chicken fibroblasts. Y. Zhang et al. reported that MSC transplantation into diabetic rats reduced blood glucose level and prevented renal damages, which was enhanced by ultrasound-targeted microbubble destruction. MSCs have low proliferative potential, which may be a hurdle for their therapeutic use. Y. Hu et al. used nonviral rDNA vectors to transfer genes into hMSCs. They found that these vectors enhanced the proliferation of hMSCs and succeeded in gene transfer. H. He et al., succeeded in differentiating BM-MSCs to hepatocytes on decellularized ECM.

When we apply regenerative medicine, we have to think that transplanted tissue must function appropriately. X.-M. Fu et al. reported that sympathetic innervation could be effectively induced into engrafted engineered cardiomyocyte sheets using GDNF. 

